# Deletion of angiotensin-converting enzyme 2 exacerbates renal inflammation and injury in apolipoprotein E-deficient mice through modulation of the nephrin and TNF-alpha-TNFRSF1A signaling

**DOI:** 10.1186/s12967-015-0616-8

**Published:** 2015-08-06

**Authors:** Hai-Yan Jin, Lai-Jiang Chen, Zhen-Zhou Zhang, Ying-Le Xu, Bei Song, Ran Xu, Gavin Y Oudit, Ping-Jin Gao, Ding-Liang Zhu, Jiu-Chang Zhong

**Affiliations:** State Key Laboratory of Medical Genomics and Shanghai Key Laboratory of Hypertension, Shanghai Institute of Hypertension, Ruijin Hospital Affiliated to Shanghai Jiao Tong University School of Medicine, 197 Ruijin 2nd Road, Shanghai, 200025 China; Pôle Sino-Français de Recherches en Science du Vivant et Génomique, Department of Mental Health, Ruijin Hospital, School of Medicine, Shanghai Jiao Tong University, Shanghai, 200025 China; Institute of Health Sciences, Shanghai Institute for Biological Sciences, Chinese Academy of Sciences, Shanghai, 200025 China; Department of Medicine, Mazankowski Alberta Heart Institute, University of Alberta, Edmonton, T6G 2S2 Canada

**Keywords:** Angiotensin-converting enzyme 2, Apolipoprotein E, Angiotensin II, Inflammation, Nephrin, Renal injury

## Abstract

**Background:**

The renin-angiotensin system (RAS) has been implicated in atherosclerotic lesions and progression to chronic kidney diseases. We examined regulatory roles of angiotensin-converting enzyme 2 (ACE2) in the apolipoprotein E (ApoE) knockout (KO) kidneys.

**Methods:**

The 3-month-old wild-type, ApoEKO, ACE2KO and ApoE/ACE2 double-KO (DKO) mice in a C57BL/6 background were used. The ApoEKO mice were randomized to daily deliver either Ang II (1.5 mg/kg) and/or human recombinant ACE2 (rhACE2; 2 mg/kg) for 2 weeks. We examined changes in pro-inflammatory cytokines, renal ultrastructure, and pathological signaling in mouse kidneys.

**Results:**

Downregulation of ACE2 and nephrin levels was observed in ApoEKO kidneys. Genetic ACE2 deletion resulted in modest elevations in systolic blood pressure levels and Ang II type 1 receptor expression and reduced nephrin expression in kidneys of the ApoE/ACE2 DKO mice with a decrease in renal Ang-(1-7) levels. These changes were linked with marked increases in renal superoxide generation, NADPH oxidase (NOX) 4 and proinflammatory factors levels, including interleukin (IL)-1beta, IL-6, IL-17A, RANTES, ICAM-1, Tumor necrosis factor-alpha (TNF-alpha) and TNFRSF1A. Renal dysfunction and ultrastructure injury were aggravated in the ApoE/ACE2 DKO mice and Ang II-infused ApoEKO mice with increased plasma levels of creatinine, blood urea nitrogen and enhanced levels of Ang II in plasma and kidneys. The Ang II-mediated reductions of renal ACE2 and nephrin levels in ApoEKO mice were remarkably rescued by rhACE2 supplementation, along with augmentation of renal Ang-(1-7) levels. More importantly, rhACE2 treatment significantly reversed Ang II-induced renal inflammation, superoxide generation, kidney dysfunction and adverse renal injury in ApoEKO mice with suppression of the NOX4 and TNF-alpha-TNFRSF1A signaling. However, rhACE2 had no effect on renal NOX2 and TNFRSF1B expression and circulating lipid levels.

**Conclusions:**

ACE2 deficiency exacerbates kidney inflammation, oxidative stress and adverse renal injury in the ApoE-mutant mice through modulation of the nephrin, NOX4 and TNF-alpha-TNFRSF1A signaling. While rhACE2 supplementation alleviates inflammation, renal dysfunction and glomerulus injury in the ApoE-mutant mice associated with upregulations of Ang-(1-7) levels and nephrin expression and suppression of the TNF-alpha-TNFRSF1A signaling. Strategies aimed at enhancing the ACE2/Ang-(1-7) actions may have important therapeutic potential for atherosclerotic renal injury and kidney diseases.

## Background

Atherosclerosis is considered one of the major risk factors for renal injury and the progression of chronic kidney diseases (CKD) to end-stage renal diseases (ESRD), which is costly and important clinical event with substantial morbidity [1–4]. Patients with CKD have a substantially increased risk for atherosclerotic cardiovascular death and total mortality and loss of renal parenchyma accelerates atherosclerosis in animal models [3, 5, 6], indicating the interplay between atherosclerosis and kidney injury. Activation of the renin-angiotensin system (RAS) has an enhanced susceptibility to atherosclerotic lesions, renal inflammation and ESRD [3, 7–11]. Angiotensin (Ang) II plays a pivotal role in perpetuating glomerular injury in kidney via the modulation of nephrin signaling, the integrity of which is crucial for the glomerular filtration barrier [12]. Ang II has direct proatherosclerotic and proinflammatory actions, whereas pharmacological blockade of the RAS has anti-atherosclerotic and antiinflammatory effects, additional and independent of systemic blood pressure [7, 13–15]. The pathophysiologic mechanisms underlying the accelerated progression of renal dysfunction in atherosclerosis status and the interactions between the RAS and nephrin signaling in atherosclerotic kidney injury remain to be fully clarified.

Angiotensin-converting enzyme 2 (ACE2) is a negative regulator of the RAS, catalyzing the conversion of Ang II to the beneficial heptapeptide Ang-(1-7), thereby counterbalancing the ACE/Ang II actions [16–18]. Genetic ACE2 deficiency accentuates vascular inflammation and atherosclerosis in the apolipoprotein E (ApoE) knockout (KO) mouse with larger vascular lesions in aortic atherosclerotic plaques [7, 11, 19]. Moreover, ACE2-deficient mice are more susceptible to renal inflammation, fibrosis and kidney injury [8, 20]. In contrast, our previous studies demonstrated that ACE2 overexpression attenuates atherosclerosis in the ApoEKO mice [21] and prevents Ang II-mediated tubulointerstitial fibrosis and renal dysfunction in hypertensive mice [8–10], suggesting beneficial effects of ACE2 on the kidneys. However, the exact roles and mechanisms of ACE2 in atherosclerotic renal injury are poorly understood. In this study, we hypothesized that ACE2 is a negative regulator of kidney inflammation, oxidative stress and renal injury in the atherosclerosis-prone ApoEKO mice. To this aim, we evaluated the influences of ACE2 deficiency and Ang II infusion on renal inflammatory factors, reactive oxygen species (ROS) production and ultrastructural changes in the ApoEKO kidneys. We also examined the regulatory effects of human recombinant ACE2 (rhACE2) on the Ang II-mediated above-mentioned outcomes.

## Methods

### Animal Preparation

The 12-week-old male ApoEKO, ACE2KO and the ApoE/ACE2 double KO (DKO) mice in a C57BL/6 background were used in these experiments as previously described [8, 14, 18]. The ApoEKO mice were randomized to deliver either Ang II (1.5 mg.kg^−1^ day^−1^) or saline (vehicle) with an osmotic minipump (model 1002, Alzet Corp, Palo Alto, CA, USA) for 2 weeks as previously described [9, 14, 18]. In a separate experiment, the saline (Vehicle)- or Ang II-infused ApoEKO mice were daily treated with placebo or rhACE2 (2 mg/kg, intraperitoneal) as before [8, 9]. Throughout the study mice were housed in pathogen-free conditions and given access to standard mouse chow and water ad libitum. Systolic blood pressure (SBP) levels of mice were measured non-invasively using the tail-cuff method. All plasma determinations were performed using a Beckman CX7 chemistry analyzer for blood urea nitrogen (BUN), creatinine (Cr), total cholesterol (CHO) and triglycerides (TG). Ang II levels in plasma and renal cortex were measured by radio-immunoassay as previously described [8]. Mice were anesthetized with ketamine (80 mg/kg) and xylaxine (10 mg/kg). All experiments were approved and performed in accordance to institutional guidelines for Canadian Council on Animal Care, the Animal Research Ethics Committee at Shanghai Jiao Tong University School of Medicine and the Guide for the Care and *the Guide for the Care and Use of Laboratory Animals* published by the US National Institutes of Health (NIH Publication No. 85-23, revised 1996).

### Transmission electron microscope analysis, immunohistological and immuno-fluorescence staining

For transmission electron microscope (TEM) analysis, samples of mouse renal cortex tissues were immediately cut into small pieces and prefixed in 2.5% glutaraldehyde as previously described [18]. The renal ultrastructure of mouse was observed on a PHILIPS CM-120 transmission electron-microscope (Holland) with ×4200 and ×7400 magnifications. Five micrometer thick formalin fixed paraffin embedded sections were used for the immuno-histological staining for AT1 receptor and the immunofluorescence staining for nephrin in the renal cortex of ApoEKO and ApoE/ACE2 DKO mice as previously described [18].

### Quantitative real-time PCR analysis

The mRNA levels were evaluated by quantitative real-time reverse transcription PCR as before [8, 16]. Total RNA was extracted from renal cortex tissues using TRIzol reagent (Invitrogen) and the cDNA was synthesized using the PrimeScript RT reagent kit (TAKARA). Sequences of the forward primers and reverse primers are listed in Table 1. The quantitative real-time PCR was run in 384-well plates using a SYBR Premix ExTaq II (TAKARA). Relative mRNA levels were quantified using ABI 7900T Real Time System SDS 2.3 software (Applied. Biosystems, Carlsbad, CA, USA). GAPDH was used as an endogenous control. All samples were run in triplicates.

**Table 1:** Primers sequences for real-time PCR analysis

Genes	Primers	Sequences (5′–3′)
TNF-α	Forward primer	5′-ACAAGGCTGCCCCGACTAC-3′
	Reverse primer	5′-TCTCCTGGTATGAGATAGCA-3′
TNFRSF1A	Forward primer	5′-CCTCCTCAGTGGGTTTCT-3′
	Reverse primer	5′-CGCCTTTCTATGCTTGTCC-3′
TNFRSF1B	Forward primer	5′-TGATGACATCGGTTGAAAG-3′
	Reverse primer	5′-TGATGACATCGGTTGAAAG-3′
IL-1β	Forward primer	5′-AACCTGCTGGTGTGTGACGTTC-3′
	Reverse primer	5′-CAGCACGAGGCTTTTTTGTTGT-3′
IL-6	Forward primer	5′-ACAACCACGGCCTTCCCTACTT-3′
	Reverse primer	5′-CACGATTTCCCAGAGAACATGTG-3′
IL-17A	Forward primer	5′-GGACTCTCCACCGCAATGA-3′
	Reverse primer	5′-GTTTCTTAGGGGTCAGCCG-3′
NOX2	Forward primer	5′-TTGTGGGAGACTGGACGGA-3′
	Reverse primer	5′-ATGGAGGCAAAGGGCGTGA-3′
NOX4	Forward primer	5′-TCTCCATTGCCCCAGTGTA-3′
	Reverse primer	5′-AGGCAGTAGCAAATCCCG-3′
Nephrin	Forward primer	5′-CCCATTCAAAGGCTCCGCT-3′
	Reverse primer	5′-ACTGCCCGCACTTGCTCTC-3′
GAPDH	Forward primer	5′-TGCGACTTCAACAGCAACTC-3′
	Reverse primer	5′-ATGTAGGCCATGAGGTCCAC-3′

*TNFα* tumor necrosis factor-α, *TNFRSF1A* tumor necrosis factor receptor superfamily member 1A, *TNFRSF1B* tumor necrosis factor receptor superfamily member 1B, *IL-1β* interleukin-1β, *IL-6* interleukin-6, *IL-17A* interleukin-17A, *NOX2* NADPH oxidase 2, *NOX4* NADPH oxidase 4, *GAPDH* glyceraldehyde-3-phosphate dehydrogenase.

### Cytokine protein arrays and western blotting analysis

To further implore the effects of ACE2 deficiency on inflammation in ApoEKO mice, we studied the renal levels of pro-inflammatory cytokines/chemokines by using the RayBio^®^ C-Series Mouse Cytokine Antibody Array 1000 (Array C3 and C4; RayBiotech Inc, Norcross, GA, USA; http://www.raybiotech.com/c-series-mouse-cytokine-array-c1000-2.html). The cytokine protein arrays were performed according to manufacturer’s instructions as previously described [22]. Biotin-conjugated immunoglobulin G served as a positive control at six spots. For each sample spot, the ratio of relative expression was established after subtraction of the background intensity and comparison with the positive spots available in the membrane. The proteins from renal cortex tissues were measured by Western blotting analysis as previously described [9, 17]. The primary antibody against ACE2 (90 kD), nephrin (185 kD), tumor necrosis factor-α (TNF-α; 17 kD), TNFRSF1A (55 kD), IL-17A (17 kD), AT1 (41 kD) and β-actin (45 kD) were obtained from R&D Systems (Minneapolis, MN), Abcam Inc. (Cambridge, MA,USA), Cell Signaling Technology (Beverly, MA), and Santa Cruz Biotechnology (Santa Cruz, CA, USA), respectively. β-actin was used as an endogenous control.

### Dihydroethidium fluorescence and measurement of NADPH oxidase activity

To evaluate superoxide production in kidneys of mice, we performed the oxidative fluorescent dye dihydroethidium (DHE) staining as previously described [8, 18]. Briefly, fresh frozen tissue sections (20 μm) for mouse kidneys were incubated at 37°C for 30 min with DHE (20 μM) in hanks balanced salt solution. For a separated experiment, kidney tissue sections from the ApoEKO mice with Ang II pumps were incubated with polyethylene glycol-conjugated superoxide dismutase (PEG-SOD; 500 U/mL) at 37°C for 30 min prior to 30-min exposure of DHE (20 μM). Fluorescent images were observed with an Olympus Fluoview laser-scanning confocal microscope mounted on an Olympus microscope selected with CY3 (red) channel. The activities of nicotinamide adenine dinucleotide phosphate (NADPH) oxidase in kidney issues of mice were measured by the lucigenin-enhanced chemiluminescence assay as previously reported [8, 18]. The kidney homogenates (100 μg total proteins) were collected in 100 μl of PBS mixture with protease and phosphatase inhibitor (Sigma-Aldrich) and then centrifuged at 1,000 g for 10 min. NADPH (1 mM) and lucigenin (50 μM) were added to the supernatants for NADPH oxidase activities assay at 37°C. The diphenylene iodonium (DPI, 10 μM) was used as a selective inhibitor of NADPH oxidase. The light emission over a 3-min period was averaged for each sample.

### Statistical analysis

All data are shown as mean ± SEM. All statistical analyses were performed with SPSS software (version 16.0) either by Student’s t test for comparison between two groups or by ANOVA followed by the Student-Neuman–Keuls test for multiple-comparison testing as appropriate. A value of *P* < 0.05 was considered to indicate statistical significance.

## Results

### Deletion of ACE2 facilitates renal dysfunction in the ApoE/ACE2 DKO mice with decreased nephrin levels

**Fig. 1:**
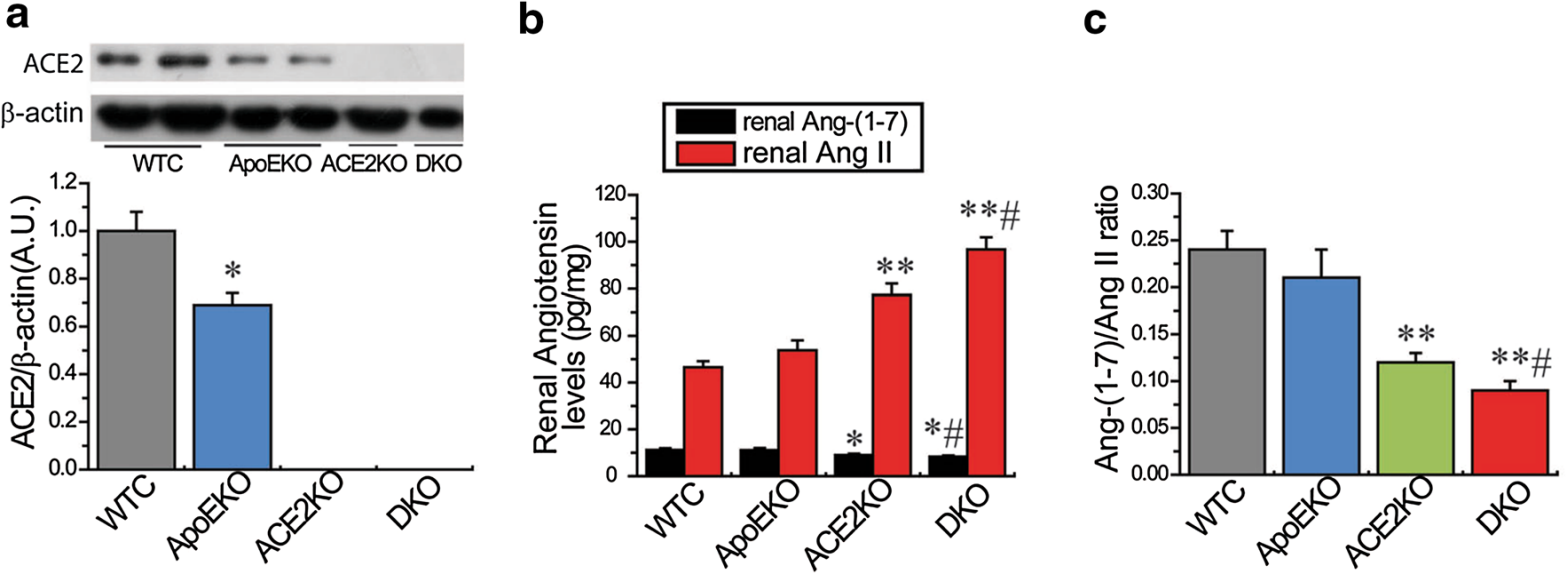
Renal ACE2, Ang II and Ang-(1-7) levels in the mice. **a** Representative Western blot analysis showing ACE2 protein levels in the mice kidneys. β-actin was used as an endogenous control. **b**, **c** The Ang II and Ang-(1-7) levels (**b**) and the ratio of Ang-(1-7)/Ang II (**c**) in the kidney cortex of mice. *AU* arbitrary units, *WT* wildtype, *ApoE* apolipoprotein E, *KO* knockout, *DKO* the ApoE/ACE2 double knockout, *Ang* *II* angiotensin II. n = 5–6. *P < 0.05;**P < 0.01 compared with WT group; ^#^ *P* < 0.05, compared with ApoEKO group.

**Fig. 2:**
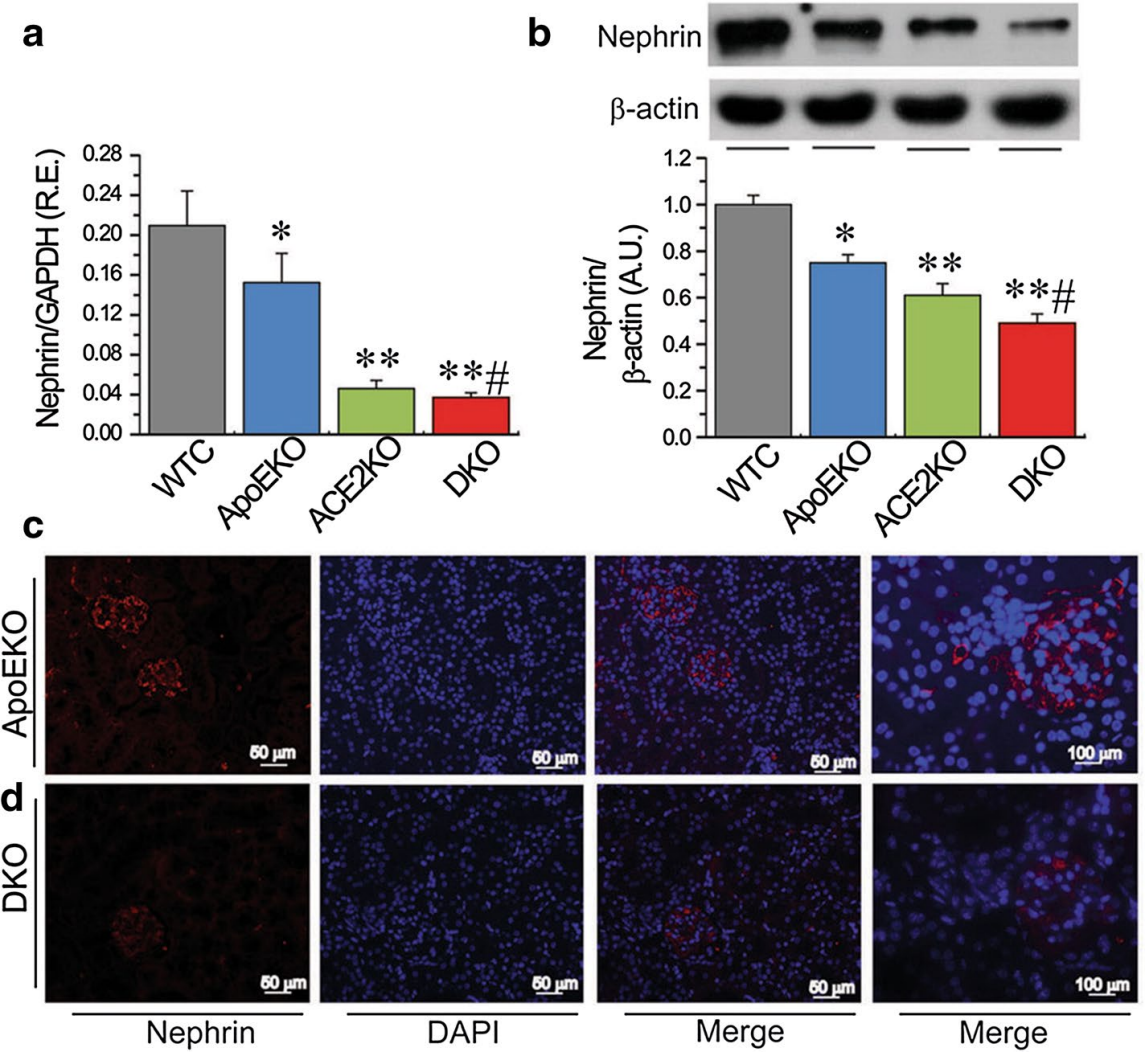
Loss of ACE2 resulted in downregulation of renal nephrin levels in the ApoE/ACE2 DKO mice. The real-time PCR and Western blot analyses for nephrin mRNA (**a**), nephrin protein (**b**) and representative nephrin immunofluorescence images (**c**, **d**) showing downregulation of nephrin levels in the ApoE/ACE2 DKO kidneys compared with the ApoEKO kidneys. n = 5–6; β-actin or GAPDH was used as an endogenous control. In the immunofluorescence images, the *red color* represents nephrin and *blue color* represents DAPI stained nuclei. *RE* relative expression, *AU* arbitrary units, *WTC* wild-type control, *ApoE* Apolipoprotein E, *KO* knockout, *DKO* the ApoE/ACE2 double knockout mice. *P < 0.05; **P < 0.01 compared with WT control group; ^#^ *P* < 0.05 compared with ApoEKO group.

Kidneys obtained from the ApoEKO mice showed marked decreases in ACE2 protein (Fig. 1) and nephrin levels (Fig. 2) compared with the kidneys from WT controls. There were no changes in SBP, Ang II and Ang-(1-7) levels between the WT and ApoEKO mice (Table 2; Fig. 1). As shown in Table 2, plasma total CHO and TG concentrations were elevated in both the ApoEKO and ApoE/ACE2 DKO mice when compared to WT control mice. However, ACE2 deficiency had no effect on circulating lipid levels in the ACE2KO mice and ApoE/ACE2 DKO mice. In addition, ACE2 deficiency led to marked down-regulations in nephrin mRNA and protein levels in the single ACE2KO and ApoE/ACE2 DKO kidneys (Fig. 2). Intriguingly, ACE2 deficiency resulted in modest elevations in SBP levels and increased renal Ang II levels in both the single ACE2-mutant and ApoE/ACE2 double-mutant mice with accompanying decreases in renal Ang-(1-7) levels and the Ang-(1-7)/Ang II ratio (Table 2; Fig. 1). These changes were associated with exacerbation of renal dysfunction with enhanced plasma levels of Cr and BUN (Table 2). Clearly, these observations revealed detrimental effect of ACE2 deficiency on nephrin signaling and renal dysfunction is dependent on the balance between Ang II and Ang-(1-7) levels.

**Table 2:** The general data in mice

	WTC	ApoEKO	ACE2KO	DKO	ApoEKO + rhACE2	ApoEKO + Ang II	ApoEKO + Ang II +rhACE2
n	8	7	8	7	7	6	5
SBP (mm Hg)	100.8 ± 3.5	103.1 ± 2.4	118.3 ± 3.1**	124.5 ± 3.3**^#^	104.5 ± 3.0	153.2 ± 4.2^##^	127.2 ± 3.1^ϕ^
BW (g)	25.1 ± 1.2	25.9 ± 1.0	24.7 ± 0.8	23.5 ± 1.2	26.1 ± 1.1	24.6 ± 1.4	25.3 ± 1.5
BUN (mmol/L)	6.4 ± 0.3	9.3 ± 0.4*	9.4 ± 0.7*	13.9 ± 0.9**^##^	8.9 ± 0.6	15.6 ± 0.7^##^	10.5 ± 0.6^ϕ^
Cr (μmol/L)	11.1 ± 0.8	18.4 ± 1.5*	19.8 ± 1.9*	40.4 ± 4.0**^##^	10.3 ± 1.0^#^	52.4 ± 7.2^##^	25.8 ± 1.7^ϕ^
TG (mmol/L)	0.59 ± 0.05	1.14 ± 0.07*	0.73 ± 0.08	1.23 ± 0.13^*^	1.06 ± 0.11	1.23 ± 0.16	1.15 ± 0.13
CHO (mmol/L)	1.48 ± 0.11	6.46 ± 0.34**	1.61 ± 0.15	7.31 ± 0.60**	6.4 ± 0.4	8.57 ± 0.43	8.25 ± 0.5
Plasma Ang II (pg/mL)	38.6 ± 1.8	43.5 ± 2.1	69.0 ± 3.8*	75.3 ± 4.6**^#^	40.9 ± 2.1	302.9 ± 15.9^##^	172.2 ± 14.4^ϕ^

*SBP* systolic blood pressure, *BW* body weight, *BUN* blood urea nitrogen, *Cr* creatinine, *TG* triacylglycerol, *CHO* cholesterol, *Ang II* angiotensin II, *KO* knockout, *WTC* wild-type control mice, *ApoE* apolipoprotein E, *DKO* ApoE/ACE2 double knockout mice, *ACE2* angiotensin-converting enzyme 2, *rhACE2* recombinant human ACE2.

Results are presented as mean ± SEM. * *P* < 0.05, ** *P* < 0.01 compared with WTC group; ^#^ *P* < 0.05, ^##^ *P* < 0.01 compared with ApoEKO control group; ^ϕ^ *P* < 0.05 compared with ApoEKO + Ang II group.

### Deletion of ACE2 facilitates renal inflammation and oxidative stress in the ApoE-deficient mice via activation of the TNF-α-TNFRSF1A and NOX4 signaling

**Fig. 3:**
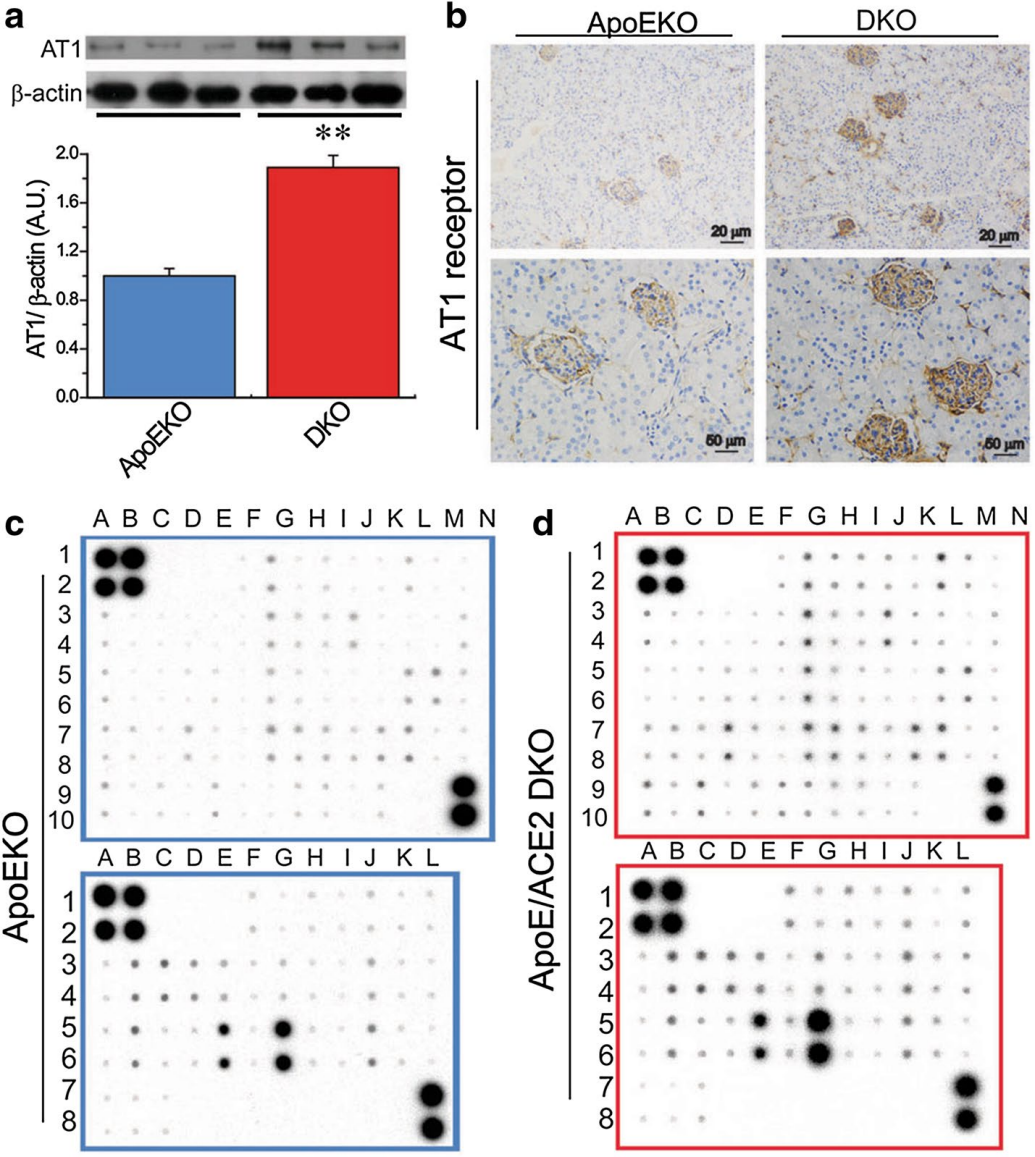
ACE2 deficiency resulted in elevated AT1 receptor levels and renal inflammation in ApoE/ACE2 DKO kidneys. **a**, **b** The Western blot analysis for AT1 receptor protein (**a**) and representative immunohistological staining images for AT1 receptor (**b**) showing upregulation of AT1 receptor levels in the ApoE/ACE2 DKO kidneys compared with the ApoEKO kidneys. n = 5; β-actin was used as an endogenous control. **c**, **d** Quantification of cytokines in kidney tissues of the ApoEKO and ApoE/ACE2 DKO mice by the mouse cytokine-specific antibody arrays (array C3: *upper*; array C4: *bottom*). n = 3 for each group. Please see the related description in Table 3. *AU* arbitrary units, *ApoE* apolipoprotein E, *KO* knockout, *DKO* the ApoE/ACE2 double knockout. **P < 0.01 compared with ApoEKO group.

**Fig. 4:**
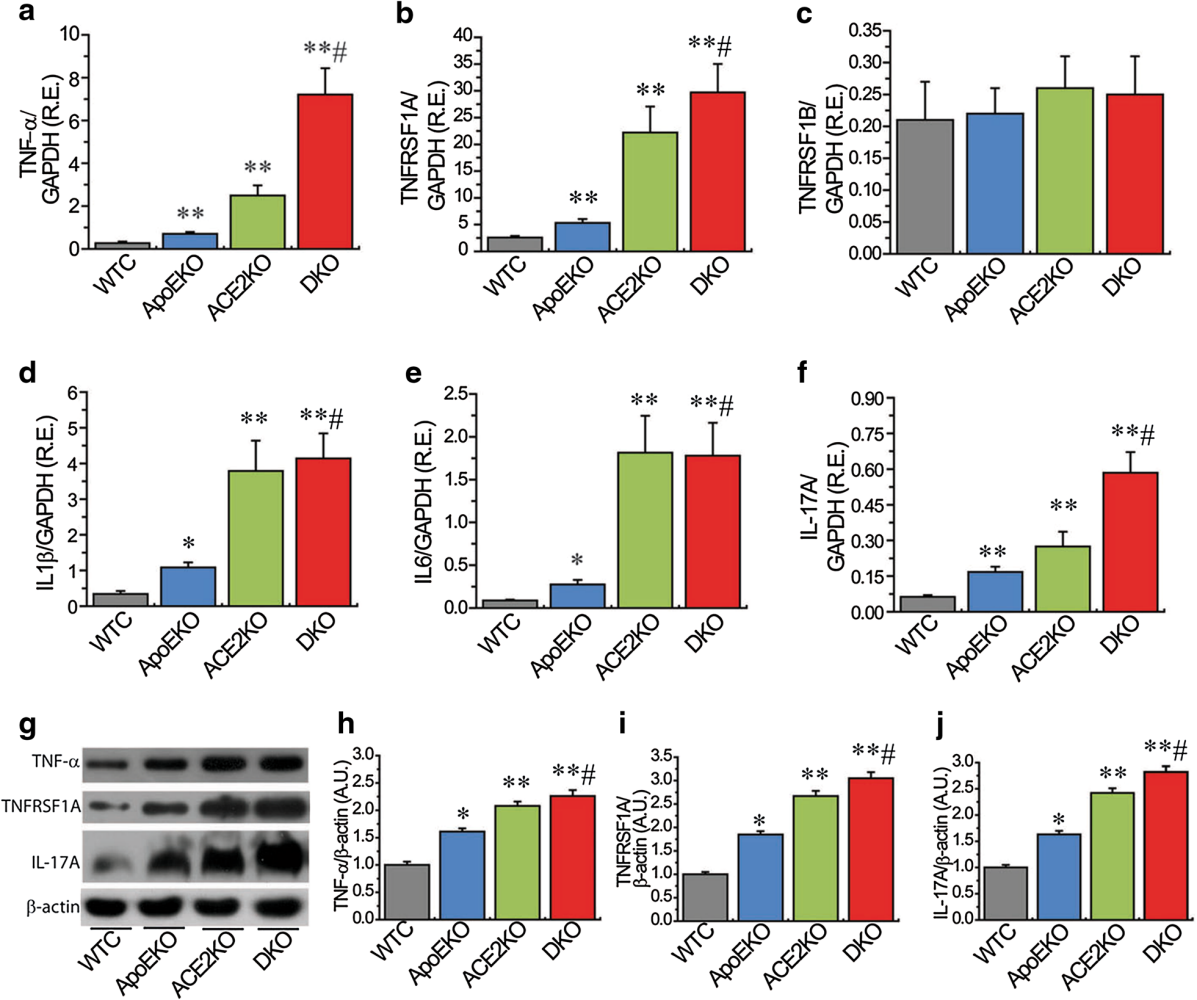
ACE2 deficiency led to increases in renal inflammation in the ApoE/ACE2 DKO mice. The real-time PCR (**a**–**f**) and Western blot analyses (**g**–**j**) revealed the mRNA or protein levels of the inflammatory cytokines in mice kidneys, including TNFα, TNFRSF1A, TNFRSF1B, IL-1β, IL-6, and IL-17A. n = 5–6. GAPDH or β-actin was used as an endogenous control. *RE* relative expression, *WTC* wild-type control, ApoE apolipoprotein E, *KO* knockout, *DKO* the ApoE/ACE2 double knockout, *TNF*α tumor necrosis factor-α, *IL* interleukin. Please see other abbreviations in Table 3. *P < 0.05; **P < 0.01 compared with WT control group; ^#^ *P* < 0.05 compared with ApoEKO group.

**Fig. 5:**
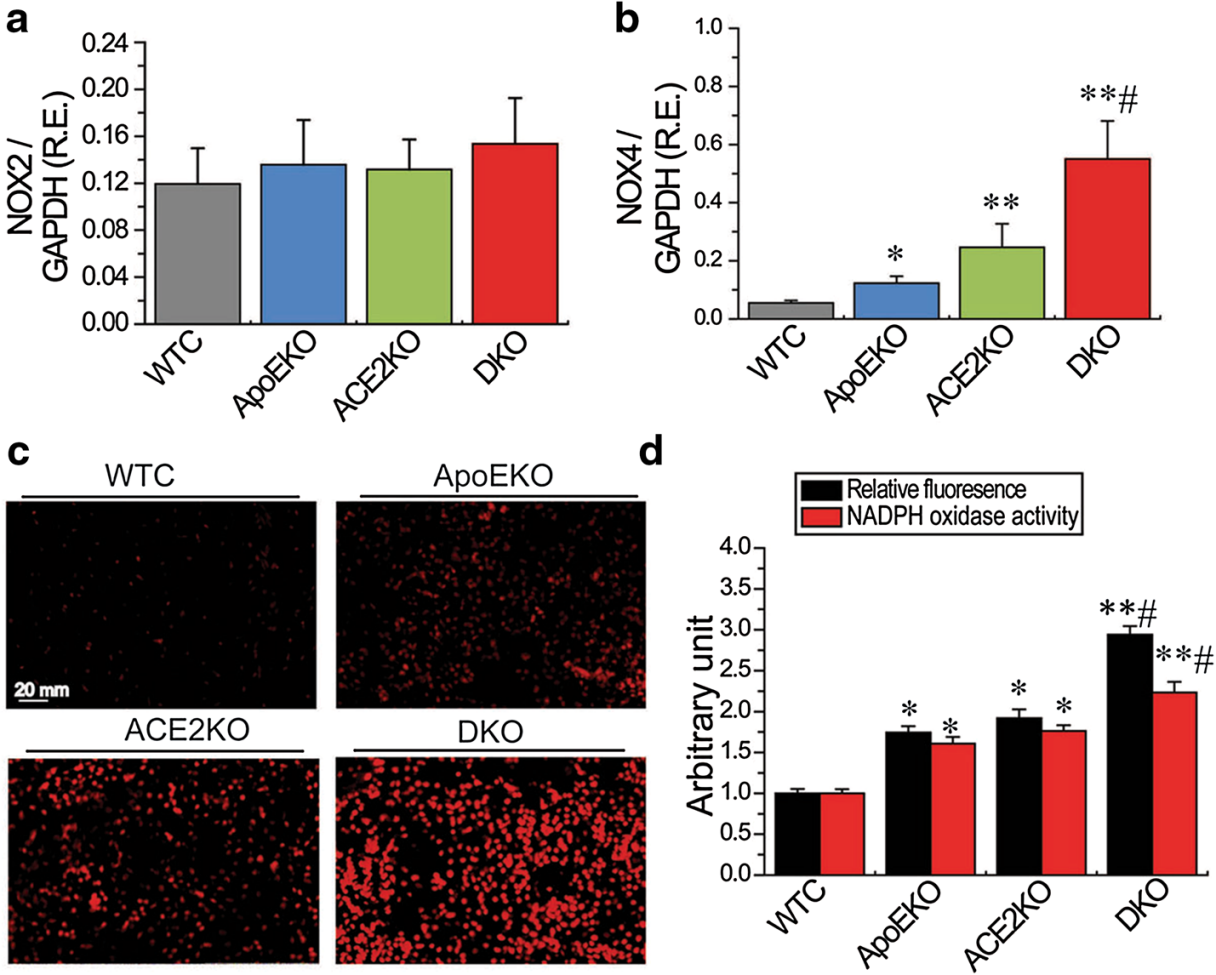
ACE2 deficiency resulted in increases in oxidative stress levels in the ApoE/ACE2 DKO kidneys. **a**, **b** The real-time PCR analysis revealed mRNA expression of the NADPH oxidase subunits NOX2 (**a**) and NOX4 (**b**) in mice kidneys (n = 6). GAPDH was used as an endogenous control. **c**, **d** Representative dihydroethidium fluorescence images (**c**), relative fluorescence values and lucigenin-enhanced chemiluminescence assay (**d**) exhibited the superoxide generation and NADPH oxidase activity in mice kidneys. n = 5. *WTC* wild-type control, *ApoE* apolipoprotein E, *ACE2* angiotensin-converting enzyme 2, *KO* knockout, *DKO* the ApoE/ACE2 double knockout. *P < 0.05; **P < 0.01 compared with WT control group; ^#^ *P* < 0.05 compared with ApoEKO group.

Activation of the RAS has been linked to inflammation and oxidative stress, key determinants of adverse renal injury [8, 20]. We evaluated the effects of ACE2 deficiency on renal inflammation and ROS levels in the ApoE/ACE2 DKO mice. The Western blot analysis and representative immunohistological staining images showed that there was upregulation of AT1 receptor levels in the ApoE/ACE2 DKO kidneys compared with the ApoEKO kidneys (Fig. 3). These changes were linked with downregulated levels of anti-inflammatory cytokine interleukin 4 (IL4) and increased levels of pro-inflammatory cytokines/chemokines and adhesion molecules in the ApoE/ACE2 DKO kidneys by cytokine protein arrays, including TNF-α, CD30L, RANTES (CCL5), intercellular adhesion molecule 1 (ICAM-1), IL-1β, IL-3, IL-6, IL-13, and IL-17A (Fig. 3; Table 3). Furthermore, ACE2 deficiency resulted in marked increases in TNF-α related signaling such as Fas Ligand (TNFSF6), GITR (TNFRSF18), Osteoprotegerin (TNFRSF11B), TRANCE (TNFSF11) and TNFRSF1A (Fig. 3; Table 3). However, there were no alterations in renal levels of TNFRSF1B, TROY (TNFRSF19), CD30, IL-1α, IL-2, IL-5, IL-7, IL-9, IL-10, IL-15, IL-17RB and VCAM-1 between the ApoEKO mice and ApoE/ACE2 DKO mice (Fig. 3; Table 3). Real-time PCR and Western blot analyses revealed that there were significant increases in expression of TNF-α, TNFRSF1A, IL-1β, IL-6, and IL-17A in the ApoEKO kidneys compared with WT control kidneys (Fig. 4). Moreover, deletion of ACE2 resulted in activation of the TNF-α-TNFRSF1A signaling and elevated levels of IL-1β, IL-6 and IL-17A in both the ACE2KO and ApoE/ACE2 DKO kidneys, without affecting the expression of TNFRSF1B (Fig. 4). Kidneys obtained from the ApoEKO mice showed elevated superoxide production (Fig. 5c, d) compared with the kidneys from WT controls. Of note, ACE2 deficiency led to increases in renal oxidative stress levels and NADPH oxidase activity (Fig. 5d) and enhanced renal expression of oxidative stress-inducible gene NADPH oxidase 4 (NOX4; Fig. 5b) not only in the single ACE2 mutant mice but also in mice carrying mutations in both ACE2 and ApoE gene (n = 5–6, *P* < 0.05, respectively). However, deletion of ACE2 had no effects on the renal NOX2 levels (Fig. 5a).

**Table 3:** Renal protein levels of cytokines and TNF-α-related signaling in the ApoE/ACE2 DKO mice

Gene name	Description	Folds up- or down-regulation (vs. ApoEKO mice)	*P* value
TNF-α	Tumor necrosis factor	5.16	0.023
CD30 ligand	TNFSF8; TNF ligand superfamily member 8	1.92	0.012
CD30	TNFRSF8; TNF receptor superfamily member 8	1.15	0.540
Fas ligand	TNFSF6; TNF ligand superfamily member 6	2.74	0.025
TNFRSF1A	TNF RI; TNF receptor superfamily member 1A	2.10	0.020
TNFRSF1B	TNF RII; TNF receptor superfamily member 1B	1.09	0.665
GITR	TNFRSF18; TNF receptor superfamily member 18	2.28	0.026
OGN	Osteoprotegerin; TNF receptor superfamily member 11B	2.27	0.024
TRANCE	TNFSF11;TNF ligand superfamily member 11	2.15	0.023
TROY	TNFRSF19; TNF receptor superfamily member 19	1.19	0.456
RENTES	CCL5; C–C motif chemokine 5	2.33	0.006
ICAM-1	Intercellular adhesion molecule 1	1.79	0.014
VCAM-1	Vascular cell adhesion protein 1	1.14	0.730
IL-1α	Interleukin-1 alpha	1.47	0.205
IL-1β	Interleukin-1 beta	2.69	0.025
IL-2	Interleukin-2	1.80	0.101
IL-3	Interleukin-3	2.34	0.034
IL-4	Interleukin-4	−1.74	0.024
IL-5	Interleukin-5	1.41	0.567
IL-6	Interleukin-6	2.61	0.033
IL-7	Interleukin-7	1.37	0.293
IL-9	Interleukin-9	1.62	0.220
IL-10	Interleukin-10	−1.19	0.065
IL-2p40/p70	Interleukin-12 subunit beta	1.71	0.154
IL-12 p70	Interleukin-12 subunit alpha	1.20	0.539
IL-13	Interleukin-13	2.43	0.023
IL-15	Interleukin-15	1.38	0.183
IL-17A	Interleukin-17A	3.11	0.010
IL-17RB	Interleukin-17 receptor B	1.22	0.301

### Treatment with rhACE2 prevents renal dysfunction and inflammation in the ApoEKO mice in response to Ang II

**Fig. 6:**
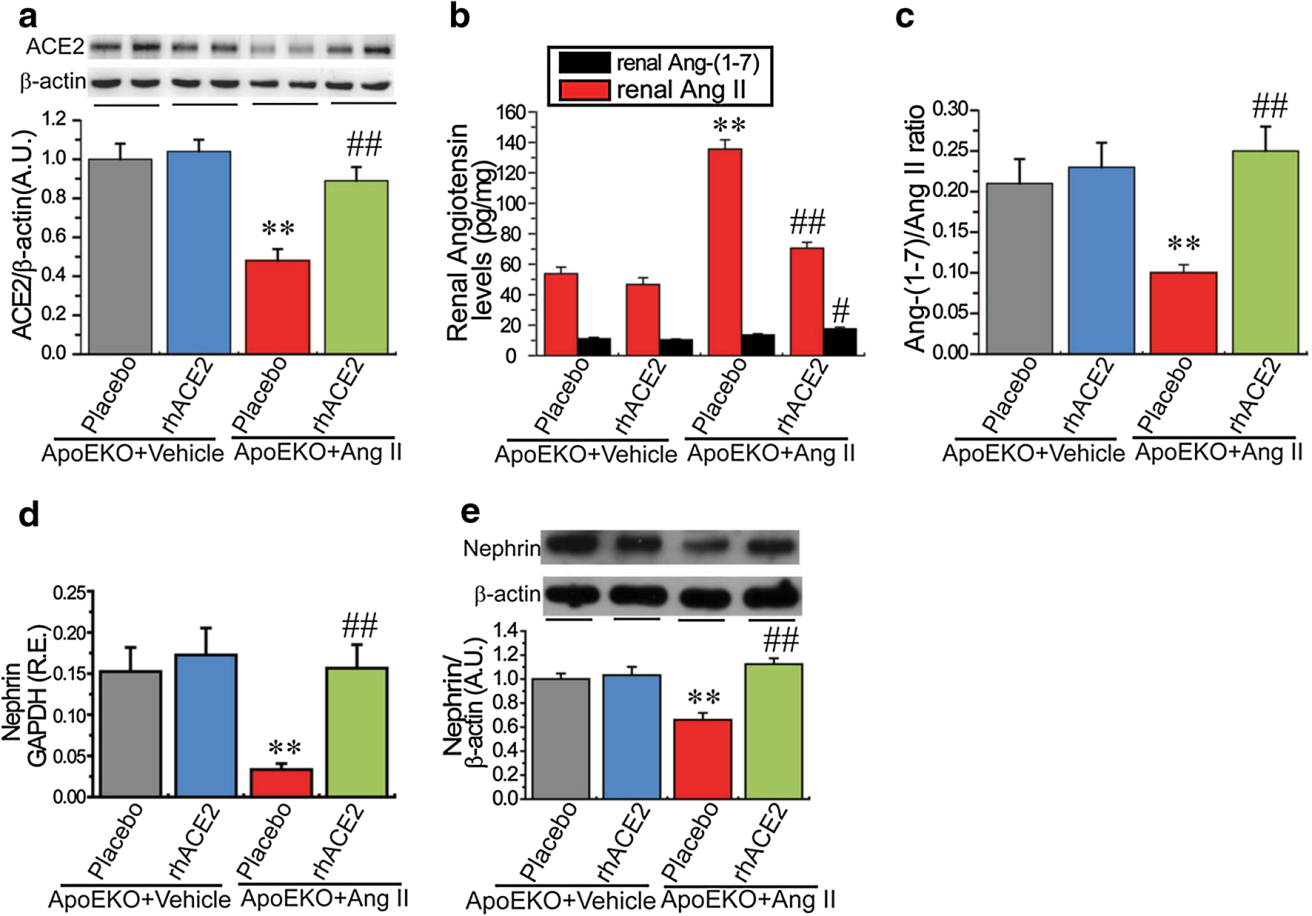
Treatment with rhACE2 abolished Ang II-mediated reduction in renal nephrin expression in ApoEKO mice. **a** Representative Western blot analysis showing ACE2 protein levels in the mice kidneys. **b**, **c** The Ang II and Ang-(1-7) levels (**b**) and the ratio of Ang-(1-7)/Ang II (**c**) in the kidney cortex of the ApoEKO mice treated with Ang II and/or human recombinant ACE2 (rhACE2). **d**, **e** The real-time PCR (**d**) and Western blotting analyses (**e**) exhibited levels of nephrin in mice kidneys. β-actin or GAPDH was used as an endogenous control. n = 5–6; *RE* relative expression, *AU* arbitrary units; **P < 0.01 compared with ApoEKO control group; ^#^ *P* < 0.05; ^##^ *P* < 0.01 compared with ApoEKO + Ang II group.

**Fig. 7:**
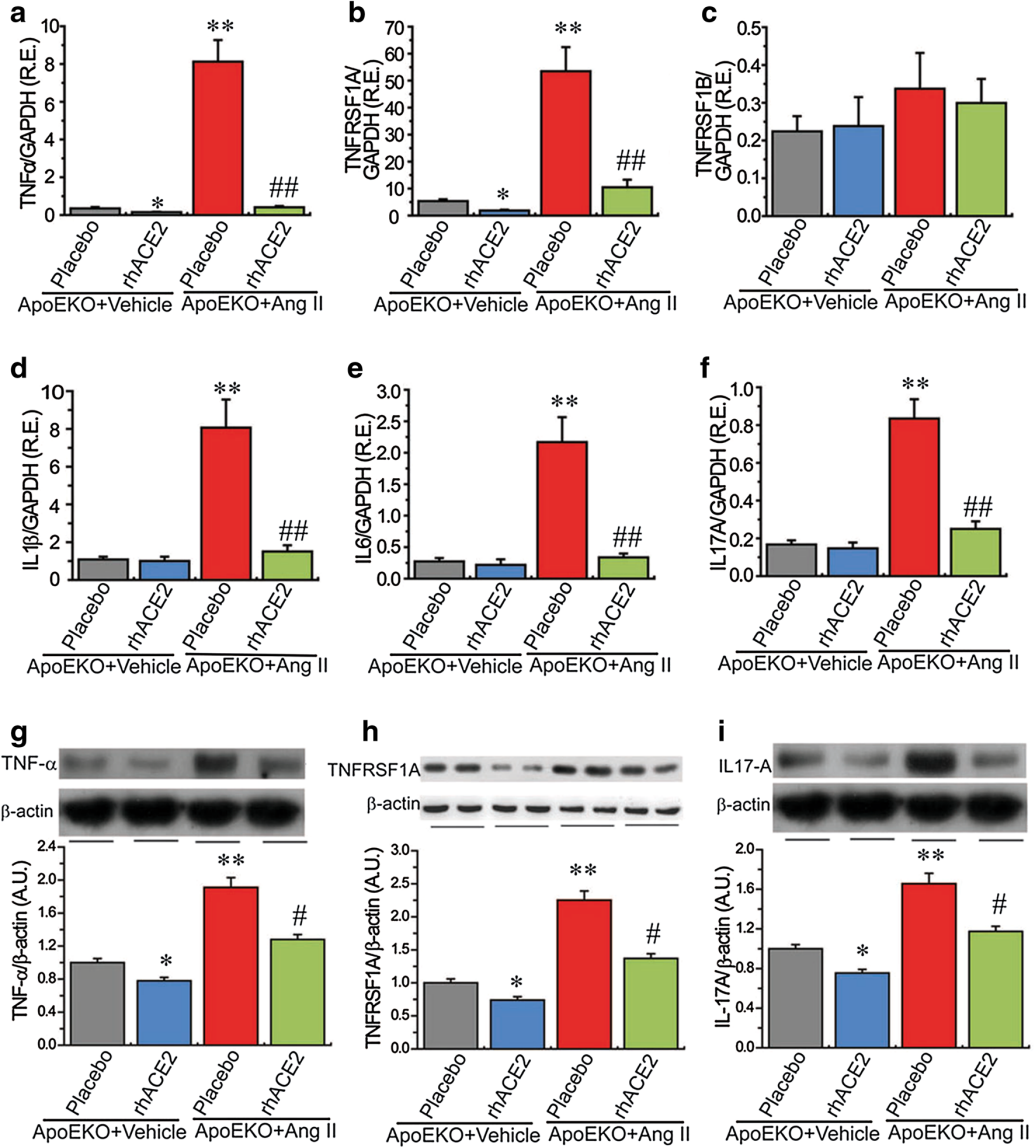
Treatment with rhACE2 prevented Ang II-mediated renal inflammation in the ApoEKO mice. The real-time PCR (**a**–**f**) and Western blot analyses (**g**–**i**) demonstrated the renal mRNA or protein expression of inflammatory cytokines in the ApoEKO mice, including TNFα, TNFRSF1A, TNFRSF1B, IL-1β, IL-6, and IL-17A (n = 4–6). GAPDH or β-actin was used as an endogenous control. *RE* relative expression, *rhACE2* human recombinant ACE2, *Ang II* angiotensin II. Please see other abbreviations in Table 3. *P < 0.05; **P < 0.01 compared with ApoEKO control group; ^#^ *P* < 0.05; ^##^ *P* < 0.01 compared with ApoEKO + Ang II group.

Given that ACE2 deficiency is functionally relevant in worsening the impaired kidney phenotypes of the ApoEKO mice, we speculated that rhACE2 supplementation should rescue Ang II-mediated pathological actions in kidneys. In response to Ang II, the renal ACE2 protein and renal Ang-(1-7)/Ang II ratio were markedly decreased in the ApoEKO mice with elevated Ang II levels in kidneys and plasma (Table 2; Fig. 6). There was no significant change in Ang-(1-7) levels between the vehicle-treated ApoEKO and Ang II-treated ApoEKO mice (Fig. 6). Continuous infusion of Ang II resulted in a predicted pressor response, a reduction in nephrin levels and exacerbation of renal dysfunction in the ApoEKO mice associated with enhanced plasma levels of Cr and BUN (Table 2; Fig. 6). Intriguingly, treatment with rhACE2 rescued Ang II-induced hypertension and renal dysfunction in the Ang II-infused ApoEKO mice associated with down-regulation of Ang II levels (Table 2; Fig. 6). The Ang II-mediated reductions of renal ACE2 and nephrin levels in the ApoE-mutant mice were significantly prevented by rhACE2 supplementation, along with an increase in renal Ang-(1-7)/Ang II ratio (Fig. 6). However, in the vehicle-infused ApoEKO mice, rhACE2 treatment significantly reduced plasma Cr levels without affecting SBP, BUN, Ang II and Ang-(1-7) levels (Table 2). Administration of rhACE2 had no effects on circulating levels of TG and CHO in both the vehicle-infused and Ang II-infused ApoEKO mice (Table 2). Consistent with worsened renal dysfunction, Ang II infusion in the ApoEKO mice exhibited exacerbation of kidney inflammation with enhanced expression of TNF-α, TNFRSF1A, IL-1β, IL-6, and IL-17A (Fig. 7). Treatment with rhACE2 reduced renal levels of TNF-α and TNFRSF1A, but not TNFRSF1B, in both the vehicle-infused and Ang II-infused ApoEKO mice (Fig. 7). Notably, rhACE2 supplementation was effective at suppressing the increased expression of IL-1β, IL-6, and IL-17A in the Ang II-infused ApoEKO mice (Fig. 7). Taken together, these data provide some evidence for protective roles of ACE2 in the Ang II-mediated renal dysfunction and inflammation in the ApoEKO mice through normalization of the nephrin and TNF-α-TNFRSF1A signaling.

### ACE2 is a negative regulator in kidney superoxide generation and adverse renal injury in the ApoE-deficient mice

**Fig. 8:**
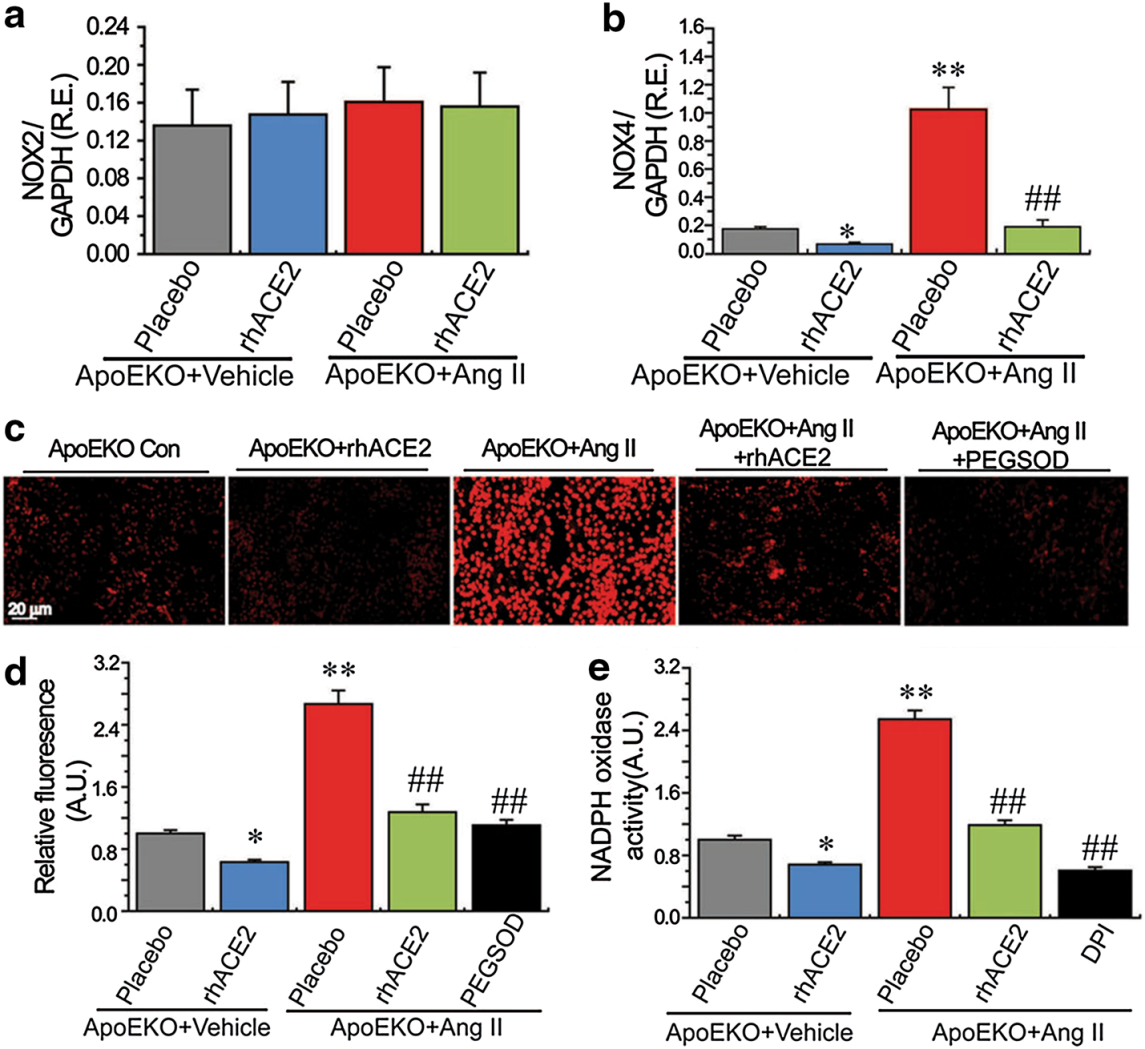
Effects of rhACE2 on renal oxidative stress levels in the Ang II-infused ApoEKO mice. **a**, **b** The real-time PCR analysis revealed mRNA expression of the NADPH oxidase subunits NOX2 (**a**) and NOX4 (**b**) in mice kidneys (n = 4–6). GAPDH was used as an endogenous control. **c**–**e** Representative dihydroethidium fluorescence images (**c**), relative fluorescence values (**d**) and lucigenin-enhanced chemiluminescence assay (**e**) exhibited the superoxide generation and NADPH oxidase activity in mice kidneys. n = 4–5. *AU* arbitrary units, *ApoE* apolipoprotein E, *ACE2* angiotensin-converting enzyme 2, *KO* knockout, *rhACE2* human recombinant ACE2, *Ang II* angiotensin II, *PEG-SOD* polyethylene glycol-conjugated superoxide dismutase, *DPI* diphenylene iodonium chloride (NADPH oxidase inhibitor). *P < 0.05; **P < 0.01 compared with the ApoEKO control group; ^##^ *P* < 0.01, compared with the ApoEKO + Ang II group.

**Fig. 9:**
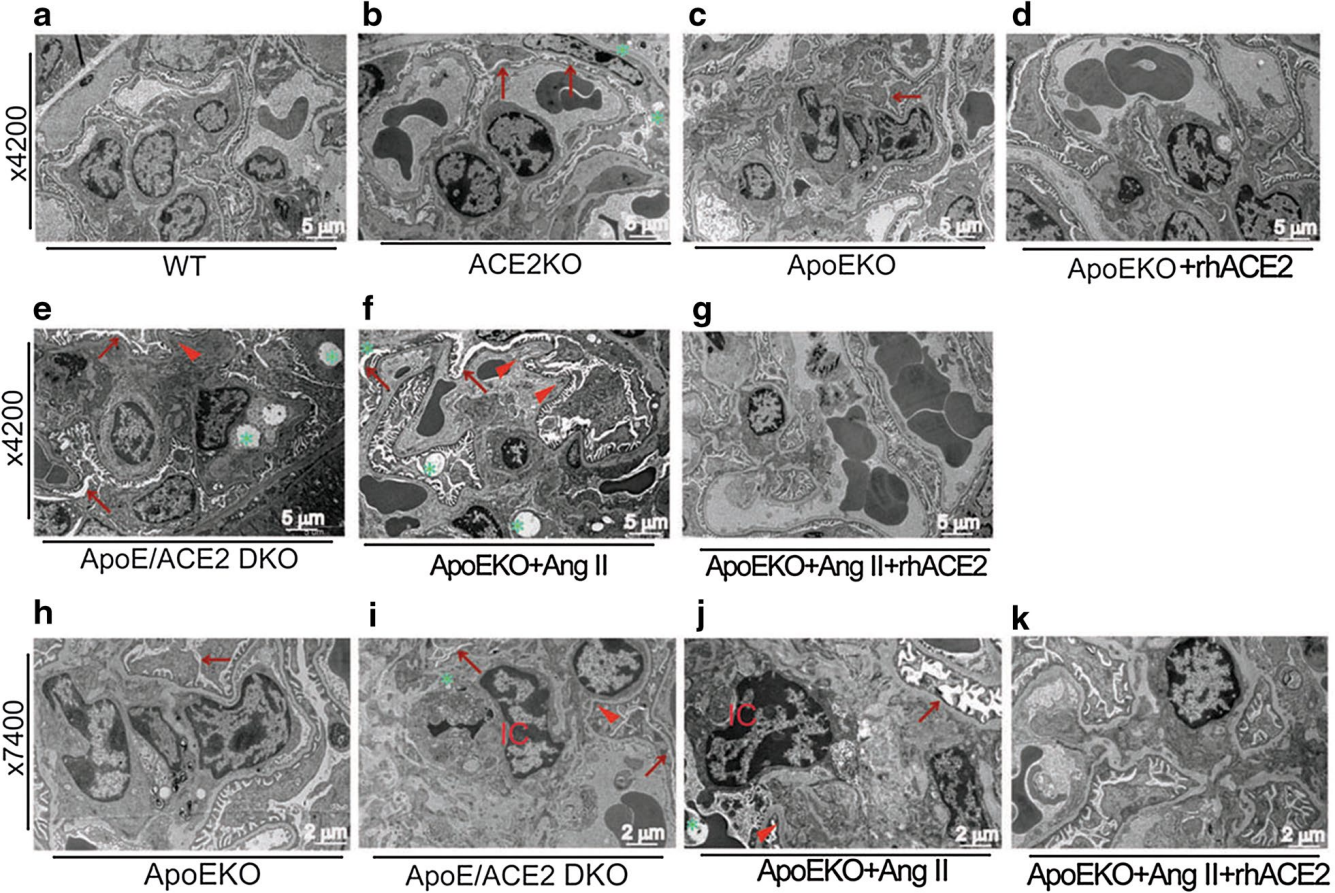
Renal ultrastructure changes in mice. The renal glomerulus ultrastructural changes were observed in kidneys of mice by transmission electron microscope analysis (**a**–**g** ×4200 magnification; **h**–**k** ×7400 magnification). Compared with the ApoEKO mice, renal ultrastructure injury was aggravated in the ApoE/ACE2 DKO mice or the Ang II-infused ApoEKO mice. These ultrastructure changes were characterized with renal mesangial cell necrosis (*green star*), the immune-complex (IC) formation, podocyte depletion from the glomerular wall linked with the foot process effacement (*red arrow*) and the thickening and stiffening glomerular capillary basement membrane (*red triangle*). *WTC* wild-type control, *ApoE* apolipoprotein E, *ACE2* angiotensin-converting enzyme 2, *KO* knockout, *DKO* double knockout, *rhACE2* human recombinant ACE2, *Ang II* angiotensin II, *IC* immune-complex.

**Fig. 10:**
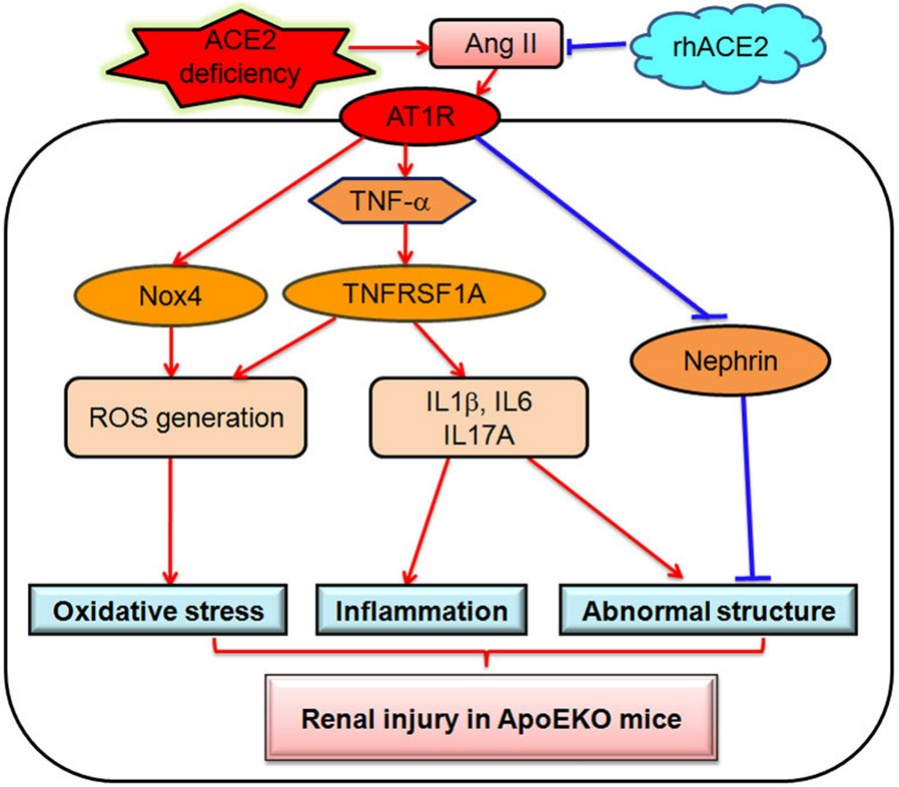
Effects and potential mechanisms of ACE2 on renal inflammation and injury in ApoE-deficient mice. ACE2 serves as a key regulator of Ang II-mediated actions in kidneys. On one hand, ACE2 deficiency results in downregulation of nephrin levels and greater increases in ROS production and expression of proinflammatory cytokines TNFα, IL-1, IL-6, and IL-17A, contributing to renal inflammation, oxidative stress and structural injury in the ApoEKO mice. On the other hand, rhACE2 treatment promotes nephrin levels and ameliorates the Ang II-induced kidney inflammation and ROS generation, functioning as a negative regulator for kidney dysfunction and renal injury in the ApoEKO mice.

We next examined the influences of rhACE2 treatment on renal oxidative stress levels and glomerulus ultrastructure changes in the ApoEKO mice. Ang II infusion resulted in a predictable increase in renal ROS production in the ApoEKO mice as assessed by DHE staining (Fig. 8c, d). Importantly, Ang II-induced increases in superoxide generation in the ApoEKO kidneys were largely rescued by rhACE2 treatment (Fig. 8c) because of prevention of the Ang II-mediated activation of NADPH oxidase (Fig. 8c) and expression of NOX4 (Fig. 8b), with no change in NOX2 levels (Fig. 8a). The superoxide scavengers, polyethylene glycol-superoxide dismutase (650 U/ml) and diphenylene iodonium (10 μmol/l) (Fig. 8), were used to confirm the Ang II-mediated ROS production in the ApoEKO kidneys. Consistent with exacerbation in renal inflammation and oxidative stress, renal glomerulus ultrastructure injury was aggravated in the ApoE/ACE2 DKO mice or Ang II-infused ApoEKO mice when compared with the ApoEKO control mice (Fig. 9). These ultrastructure changes were characterized with renal mesangial cell necrosis, podocyte depletion from the glomerular wall, the foot process effacement and the thickening glomerular capillary basement membrane (Fig. 9), contributing to progression of kidney damage. Notably, there were immune complexs, another key determinant of adverse renal injury, in glomerulus of kidneys in both the ApoE/ACE2 DKO mice and Ang II-infused ApoEKO mice (Fig. 9). In response to rhACE2 treatment, renal ultrastructure injury was alleviated in the Ang II-infused ApoEKO mice (Fig. 9). ACE2 serves as a negative regulator of Ang II-mediated abnormal nephrin signaling, inflammation and oxidative stress in the kidneys, ultimately contributing to attenuation of kidney dysfunction and improvement of adverse renal injury in the ApoEKO mice (Fig. 10).

## Discussion

The present study uncovered the insights into the involvement of ACE2 deficiency in the development of kidney inflammation, oxidative stress and adverse renal injury in the atherosclerosis-prone ApoEKO mice. To our knowledge, this is the first report of the relationship between nephrin signaling and kidney injury in the absence of ApoE and ACE2 status. Importantly, recombinant human ACE2 treatment was able to rescue Ang II-mediated pathological actions in the ApoEKO mice with improvement of renal inflammation, oxidative stress and ultrastructure injury. ACE2 has local physiological effects, especially in the kidney, turning the balance within the RAS cascade from pro-inflammatory and pro-oxidant effects to anti-inflammatory and anti-oxidant effects [23–25]. Our data showed that downregulation of renal ACE2 levels was observed in the ApoEKO mice with no changes in SBP and renal Ang II levels. Genetic ACE2 deletion resulted in significant increases in SBP levels and superoxide production and exacerbation of renal dysfunction in the ACE2KO and ApoE/ACE2 double KO mice. However, deficiency of ACE2 had no effect on circulating lipid levels in both the ACE2KO and ApoE/ACE2 double KO mice. Consistent with reports from other groups [7, 11], our findings implicated that loss of ACE2 was linked with elevated Ang II levels and augmented expression of pro-inflammatory cytokines/chemokines, including TNF-α, IL-1β, and IL-6. These changes were associated with a downregulation of renal nephrin levels and a marked increase in plasma BUN and Cr levels in the ApoE/ACE2 double KO mice. Interestingly, an obvious elevation in IL-17A levels was observed in the ApoEKO kidney, and ACE2 deficiency or Ang II infusion further increased the renal levels of IL-17A in the ApoEKO status. IL-17A is involved in many inflammatory processes by promoting Ang II-mediated hypertension and vascular dysfunction, both of which are risk factors for atherosclerosis [26]. Deletion of IL-17A has been shown to reduce inflammation and increase nitric oxide (NO) levels in the ApoE/IL-17A DKO mice with no alteration in plaque burden [26], implying the contribution of IL-17A to renal inflammation in the ApoE-mutant state or ApoE/ACE2 double mutant state. Our data point to ACE2 as an important regulator of renal inflammation and a novel target for kidney-protective therapies.

Atherosclerosis is a complex inflammatory disease characterized by derangements in the renal, metabolic and immune systems, leading to renal inflammation, oxidative stress and the development of CKD [1, 3, 4, 27]. The Ang II/AT1 receptor signaling plays a critical role in the development of atherosclerosis and renal injury by controlling inflammation, oxidative stress and immune systems [3, 8, 27]. Our data demonstrated an important role of Ang II-induced pro-inflammatory and pro-oxidant state in the ApoEKO kidneys and its ability to be modulated by ACE2. We demonstrated that deletion of ACE2 facilitated renal inflammation and ROS production in both the single ACE2-mutant mice and the ApoE/ACE2 double-mutant mice. These changes were associated with enhanced expression of oxidative stress-inducible gene NOX4, with no change in NOX2 levels. Conversely, supplementation with rhACE2 dramatically reduced the Ang II-mediated oxidative stress in the ApoEKO mice in part by preventing the expression of NOX4 and the activity of NADPH oxidase. In addition, we found that the ApoE-mutant mice lacking ACE2 (ApoE/ACE2 DKO) exhibited substantial increases in renal AT1 receptor levels and kidney inflammation as demonstrated by remarkable upregulations of proinflammatory cytokines TNF-α, IL-1β, IL-6, RANTES, ICAM-1 and TNF-α related signaling such as TNFSF6, TNFRSF18, TNFRSF11B, TNFSF11 and TNFRSF1A. Within the inflammatory response, the Ang II/AT1 receptor-induced TNF-α signaling can control the generation of anti-inflammatory cytokine NO and potentiate renal expression of pro-inflammatory cytokines IL-1 and IL-6 through the activation of nuclear factor-κB (NF-κB) signaling, thereby functioning as a major regulator of inflammation and kidney damage [27, 28]. Treating ACE2-mutant mice with TNF-α triggered upregulated expression of inflammatory factor NF-κB [7]. In turn, the activation of NF-κB signaling is an active source of pro-inflammatory cytokine TNF-α, further amplifying proinflammatory effects of the Ang II/AT1 receptor. NF-κB plays an important role in the Ang II/AT1 receptor-mediated TNF-α signaling, renal inflammation, and progression of renal disease [29, 30]. Deletion of ACE2 promoted the Ang II/AT1 receptor-stimulated NF-κB-dependent renal inflammation such as upregulation of TNF-α, IL-1β and increased macrophage and T cell infiltration in the kidneys, which were linked with an increase in the levels of phospho-IκBα and phospho-NF-κB/p65 and degradation of IκBα and NF-κB/p65, indicating the interaction between the NF-κB activation and the AT1 receptor-mediated TNF-α signaling [30]. Notably, TNF-α-deficient mice had blunted Ang II-induced hypertensive responses and reduced kidney damage in the model of Ang II-mediated kidney disease [27], confirming a role for kidney-derived TNF-α to promote Ang II/AT1 receptor-induced renal injury. The effects of TNF-α are initiated by engagement of two distinct TNF receptors, TNFRSF1A and TNFRSF1B [31, 32]. Engagement of TNFRSF1A is primarily thought to be responsible for the deleterious effects of TNF-α, whereas TNFRSF1B activation mediates protective mechanisms [31, 32]. Thus, the net effects of TNF-α on kidney injury in the ApoEKO mice may depend on the relative contribution of TNFRSF1A and TNFRSF1B signaling. As shown in this study, ACE2 deficiency triggers a marked increase in expression of pro-inflammatory factors in the ApoEKO kidneys, including TNF-α, and TNFRSF1A, without affecting the TNFRSF1B expression. ACE2 showed antiinflammatory properties by preventing expression of inflammatory cytokines [16, 24, 25] while selective inhibition of ACE2 resulted in a proinflammatory phenotype [11, 15, 25]. In this work, we observed that treatment with rhACE2 significantly blunted Ang II-mediated impairment of kidney function and enhanced renal inflammatory cytokines levels of IL-1β, IL-6, IL-17A in the ApoEKO mice with suppression of the TNF-α-TNFRSF1A signaling. Our findings indicated beneficial roles of ACE2 in the inflammatory response and kidney dysfunction, at least in part, through inhibition of the TNF-α-TNFRSF1A signaling pathway. However, we could not elucidate the extent to which TNF receptor activation affects renal inflammation and dysfunction in the absence of ApoE and ACE2 status. Additional researches are required to clarify this issue.

Another significant finding in the current study was the identification that downregulation of renal nephrin levels and enhanced expression of AT1 receptor were key mechanisms by which loss of ACE2 promoted progressive renal injury and dysfunction in the ApoE/ACE2 DKO mice. In this work, we found that genetic ACE2 deletion resulted in declined expression of nephrin and augmented levels of Ang II and AT1 receptor in the kidneys of the ApoE/ACE2 double-mutant mice, contributing to the exacerbation of kidney dysfunction and adverse renal injury. Ang II-mediated renal injury has been shown to be induced by abnormal expression and distribution of nephrin in kidney [12, 33–35]. Furthermore, Ang II facilitates renal injury through downregulation of nephrin and promotes podocyte injury indirectly by inducing cellular hypertrophy and alterations in the anionic charge of the glomerular basement membrane in the kidney [34, 35]. It is well established that nephrin is required during kidney development for the maturation of podocytes and formation of the slit diaphragm junctional complex [33–35]. Nephrin deficiency is considered a pathologic feature of glomerular injury [33, 35], and nephrin-mutant mice develops more exaggerated glomerular enlargement and increased apoptosis with mild proteinuria, foot process effacement, mesangial hypercellularity and sclerosis, and glomerular basement membrane thickening [35]. Consistent with reduction of nephrin levels and exacerbation in renal dysfunction, as shown in this study, renal glomerulus ultrastructure injury was aggravated in both the ApoE/ACE2 DKO mice and the Ang II-treated ApoEKO mice. These changes were characterized with renal mesangial cell necrosis, immune complexs formation, glomerular podocyte depletion, foot process effacement and the thickening glomerular capillary basement membrane. Notably, rhACE2 treatment was able to rescue Ang II-induced kidney dysfunction and adverse renal injury in the ApoEKO mice, in association with upregulated expression of ACE2 and nephrin. This is in agreement with previous reports demonstrating that blockade of the RAS ameliorates kidney injury in mice by enhancing levels of nephrin and preventing damage of kidney [35]. These data underscore renoprotective effects of ACE2 with the anti-inflammatory properties via modulation of the nephrin and TNF-α-TNFRSF1A signaling.

Clinical trial evidence indicates that the blockade of the RAS by ACE inhibitors or AT1 receptor blockers (ARB) has been shown to alleviate renal injury, improve renal function and reduce renal events in patients with chronic kidney diseases and ESRD [2, 5, 6, 29, 34]. ACE2 is an essential regulator in maintaining the balance between Ang II generation and Ang II degradation locally within the kidney regardless of systemic disease conditions [7, 8, 36]. In this work, downregulation of renal ACE2 protein was observed in the Ang II-infused ApoEKO mice with elevated Ang II levels in both plasma and kidney. Intriguingly, treatment with ACE inhibitors or ARB increases ACE2 expression and Ang-(1-7) levels in the kidney and cardiovascular system [9, 37, 38]. As such, enhancing ACE2 actions such as rhACE2 has emerged as a potential therapeutic strategy, functioning as an endogenous ACE inhibitor or natural ARB [36, 38–41]. Our present work and previous studies [9, 36, 38] have obviously exhibited downregulation of Ang II levels and upregulation of Ang-(1-7) levels in response to rhACE2 and are consistent with in vivo effects in healthy human volunteers whereby rhACE2 treatment lowered Ang II levels and augmented Ang-(1-7) levels [40] and in vitro effects of rhACE2 which degrades plasma Ang II into Ang-(1-7) [41]. In a murine model of Ang II-mediated cardiorenal injury, blocking Ang-(1-7) action by A779 remarkably prevented the cardiorenal protective roles of rhACE2, implying that the therapeutic effects of rhACE2 is clearly dependent on the action of Ang-(1-7) [36]. In the present work, compared with the ApoEKO control kidneys, the ratio of Ang-(1-7)/Ang II was observed in the Ang II-infused ApoEKO kidneys with no changes of Ang-(1-7) levels. Treatment of rhACE2 strikingly promoted renal Ang-(1-7)/Ang II ratio in the Ang II-infused ApoEKO mice. Our data implied that the increases in ACE2 protein and the ratio of Ang-(1-7)/Ang II mediated by rhACE2 treatment were responsible for the improvement of kidney dysfunction and the attenuation of adverse renal injury in the ApoE-null mice in response to Ang II. Thus, the development of novel strategies to interfere with ACE2/Ang-(1-7) might be a promising field for the therapeutic approach in atherosclerosis and kidney diseases.

## Conclusions

In summary, genetic ACE2 deletion resulted in greater increases in renal inflammation, superoxide generation and exacerbation of kidney dysfunction in an ApoE-deficient state with downregulation of nephrin signaling and elevation of NOX4 levels. In contrast, rhACE2 treatment appeared to show counter regulation against Ang II-mediated kidney inflammation, oxidative stress and renal injury by augmenting renal Ang-(1-7) levels, enhancing nephrin expression, and preventing the activation of NOX4 and TNF-α-TNFRSF1A signaling and these mechanisms may be involved in the therapeutic benefits of ACE2 in the ApoE-deficient mice. Our data suggest that ACE2 is a key negative modulator of atherosclerosis-related kidney inflammation, oxidative stress and adverse renal injury, and targeting the ACE2/Ang-(1-7) signaling has potential therapeutic importance for preventing and treating atherosclerotic renal injury and kidney diseases. In the future, it will be important to evaluate the exact mechanisms and antiatherogenic, and antiinflammatory responses to interventions aimed at amplifying ACE2 action directly. Future clinical studies in atherosclerosis and chronic kidney diseases are required to more precisely clarify the role of ACE2 or nephrin as a biomarker of atherosclerosis-related kidney injury.

## Abbreviations

ACE2: angiotensin-converting enzyme 2
Ang II: angiotensin II
ApoE: apolipoprotein E
ARB: AT1 receptor blockers
BUN: blood urea nitrogen
CHO: cholesterol
CKD: chronic kidney diseases
Cr: creatinine
ESRD: end-stage renal diseases
KO: knockout
ICAM-1: intercellular adhesion molecule 1
IL-1β: interleukin-1β
IL-6: interleukin-6
IL-17A: interleukin-17A
NOX2: NADPH oxidase 2
NOX4: NADPH oxidase 4
RAS: renin-angiotensin system
ROS: reactive oxygen species
SBP: systolic blood pressure
TG: triglycerides
TNF-α: tumor necrosis factor-α
TNFRSF1A: TNF receptor superfamily member 1A
TNFRSF1B: TNF receptor superfamily member 1B
